# A peer-led, school-based social network intervention for young people in the UK, promoting sexual health via social media and conversations with friends: intervention development and optimisation of STASH

**DOI:** 10.1186/s12889-023-15541-x

**Published:** 2023-04-11

**Authors:** Carrie Purcell, Lisa McDaid, Ross Forsyth, Sharon A. Simpson, Lawrie Elliott, Julia V. Bailey, Laurence Moore, Kirstin R. Mitchell

**Affiliations:** 1 Open University, 10 Drumsheugh Gardens, Edinburgh, EH3 7QJ UK; 2 School of Health and Wellbeing, University of Glasgow, Glasgow, UK; 3 Institute for Social Science Research, The University of Queensland, Long Pocket Precinct, 80 Meiers Rd, Indooroopilly, Brisbane, QLD 4068 Australia; 4 MRC/CSO Social and Public Health Sciences Unit, University of Glasgow, Clarice Pears Building, Byres Road, Glasgow, G12 8TA Scotland; 5 School of Health and Life Sciences, Department of Nursing and Community Health, Glasgow Caledonian University, Room 420 George Moore Building, Cowcaddens Road, Glasgow, G4 OBA UK; 6 University College London E-Health Unit, Royal Free Hospital, Upper Third Floor, Rowland Hill Street, London, NW3 2PF UK

**Keywords:** Intervention development, Sexual health, Young people, Co-development, 6SQuID, School-based, Social networks, Social media, Peer-led

## Abstract

**Background:**

The quality of school-based sex and relationships education (SRE) is variable in the UK. Digitally-based interventions can usefully supplement teacher-delivered lessons and positively impact sexual health knowledge. Designed to address gaps in core SRE knowledge, STASH (Sexually Transmitted infections And Sexual Health) is a peer-led social network intervention adapted from the successful ASSIST (A Stop Smoking in Schools Trial) model, and based on Diffusion of Innovation theory. This paper describes how the STASH intervention was developed and refined.

**Methods:**

Drawing on the Six Steps in Quality Intervention Development (6SQuID) framework, we tested a provisional programme theory through three iterative stages -: 1) evidence synthesis; 2) intervention co-production; and 3) adaptation - which incorporated evidence review, stakeholder consultation, and website co-development and piloting with young people, sexual health specialists, and educators. Multi-method results were analysed in a matrix of commonalities and differences.

**Results:**

Over 21 months, intervention development comprised 20 activities within the three stages. 1) We identified gaps in SRE provision and online resources (e.g. around sexual consent, pleasure, digital literacy), and confirmed critical components including the core ASSIST peer nomination process, the support of schools, and alignment to the national curriculum. We reviewed candidate social media platforms, ruling out all except Facebook on basis of functionality restrictions which precluded their use for our purposes. 2) Drawing on these findings, as well as relevant behaviour change theories and core elements of the ASSIST model, we co-developed new content with young people and other stakeholders, tailored to sexual health and to delivery via closed Facebook groups, as well as face-to-face conversations. 3) A pilot in one school highlighted practical considerations, including around peer nomination, recruitment, awareness raising, and boundaries to message sharing. From this, a revised STASH intervention and programme theory were co-developed with stakeholders.

**Conclusions:**

STASH intervention development required extensive adaptation from the ASSIST model. Although labour intensive, our robust co-development approach ensured that an optimised intervention was taken forward for feasibility testing. Evidencing a rigorous approach to operationalising existing intervention development guidance, this paper also highlights the significance of balancing competing stakeholder concerns, resource availability, and an ever-changing landscape for implementation.

**Trial registration:**

ISRCTN97369178.

**Supplementary Information:**

The online version contains supplementary material available at 10.1186/s12889-023-15541-x.

## Contributions to the literature

- Adapting existing successful interventions to new contexts and problems is crucial to implementation science. This paper details the adaptation of an anti-smoking intervention to sexual health promotion.
- While resource-intensive, the approach taken in STASH operationalised existing intervention development guidance to successfully adapt and optimise a peer-led, school-based intervention for feasibility testing.
- The rigorous process took into account context, stakeholder views, co-production, and dynamic programme theory development, to optimise the intervention for the best possible chance of real-world success.

## Background

Young people in the United Kingdom (UK) often cite school as their main source of sex education [1], but provision is variable [2], inconsistent, and can leave young people unprepared for the realities of sexual activity [3]. Young people's lives are enmeshed in powerful structures of peers, norms, and social influences, which can enhance or undermine school-based sex and relationships education (SRE). Sustainability – that is, the continued implementation over time—of school-delivered health interventions is challenging [4], meaning that alternative models need to be explored. Recognising that peer influence has potential to reinforce positive values and beliefs, and strengthen social norms that might influence sexual behaviour [5, 6], peer-led sexual health interventions for young people have been devised and evaluated. However, evidence of effectiveness remains limited and methodologically weak [5–9].

The context in which young people learn about sex is also shaped by the ever-increasing integration in their daily lives of digital media, including social networking sites, mobile applications (apps), online gaming, and video or photo communications [10]. Digital media offer a potential new route through which to support sexual health and wellbeing [11], and the popularity among young people of social networking platforms offers opportunities for sexual health promotion. While self-guided interactive digital interventions are known to improve sexual health knowledge [11–13], evidence of the effect of digital interventions on behaviour change, or of the effectiveness of social media interventions is so far also limited [11, 14–18].

It was with these considerations in mind that we initiated a study to develop and assess a novel school-based intervention which addressed limitations of existing sex education by exploring the combined potential of peer influence and social media [19]. STASH (Sexually Transmitted infections And Sexual Health), a peer-led intervention to prevent and reduce transmission of STIs and improve sexual health in secondary (age 12+) schools, built on the successful ASSIST (A Stop Smoking In Schools Trial) smoking prevention intervention. Mobilising ‘diffusion of innovation’ theory [20–23], the ASSIST model involved recruitment of ‘early adopter’ students (aged 12–13), who were deemed ‘influential’ by their peers to act as Peer Supporters, with the assumption that inviting 17.5% of a year group would result in recruitment of a ‘critical mass’ of 15% of that group. Professional trainers were used to train and support them to spread and sustain anti-smoking norms through informal peer interactions [20]. This theory-based approach aimed to improve the effectiveness and acceptability of the intervention, limit any burden on participating schools, and ultimately increase potential for widespread adoption and sustainment. A cluster randomised control trial (RCT) of ASSIST found that smoking was significantly reduced over a two-year period [24].

While ASSIST offered theoretical grounding, STASH targeted an older age group – 14 to 16 years – due to the maturity required for the task, and relevance of the topic in a period marked by increasing experimentation and initiation of sexual activities, but prior to first sexual intercourse for most. It also focused on a more sensitive issue (STI prevention), targeted a much more complex set of behaviours, and included social media as a mechanism for intervention delivery. These differences required significant adaptations to the ASSIST model and its programme theory. To guide what these adaptations should be, we drew on Medical Research Council (MRC) guidance on intervention development and evaluation, the Six Steps in Quality Intervention Development (6SQuID) framework, and on other key examples including Hawkins et al.’s approach to adapting ASSIST in the context of illicit drug use [24–29]. With varying levels of specificity, these frameworks highlighted the need for clear evidence and theoretical grounding (including an intervention theory of change or programme theory); collaboration and co-production with key stakeholders/users; processes of testing and adapting; and outcome and process evaluations.

This paper describes how the STASH intervention was developed, adapted, and optimised for a feasibility study (and potential subsequent trial), the findings of which are described elsewhere [30–32]. We detail the three stages of STASH intervention development and set out the key findings from each iterative stage namely: evidence collation and synthesis; intervention co-production; and intervention adaptation. We also discuss how this work contributed to development of the STASH programme theory and reflect on strengths and limitations of our approach. As such, this paper contributes to understanding of the effective development of peer-led sexual health interventions for young people. The intervention development process is described in line with the GUIDED intervention development reporting guidance [33].

## Methods

**Fig. 1:**
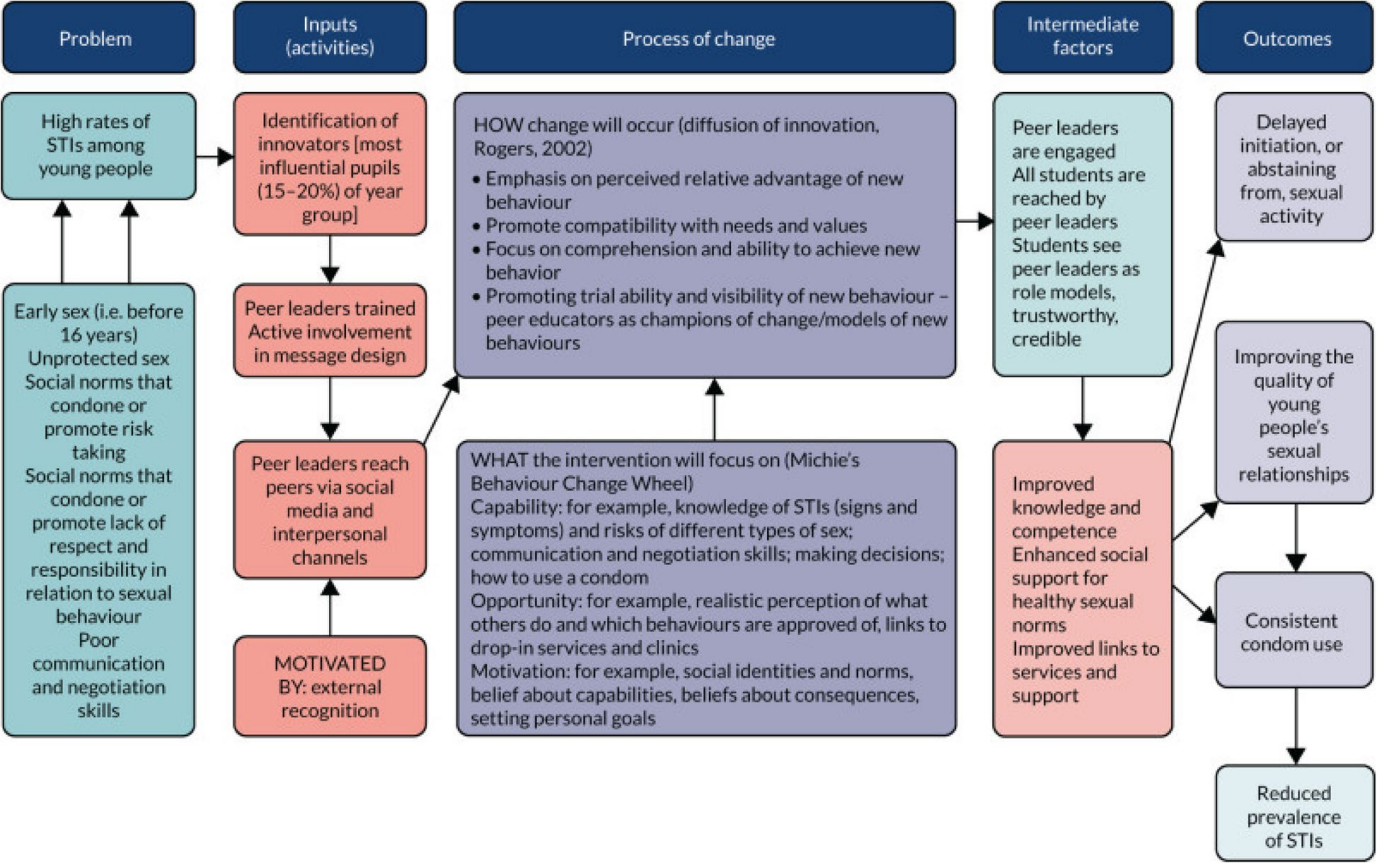
STASH Programme Theory – Proposal Version (Figures 1-4 reproduced with permission from the National Institute for Health Research (NIHR) study final report [30])

**Fig. 2:**
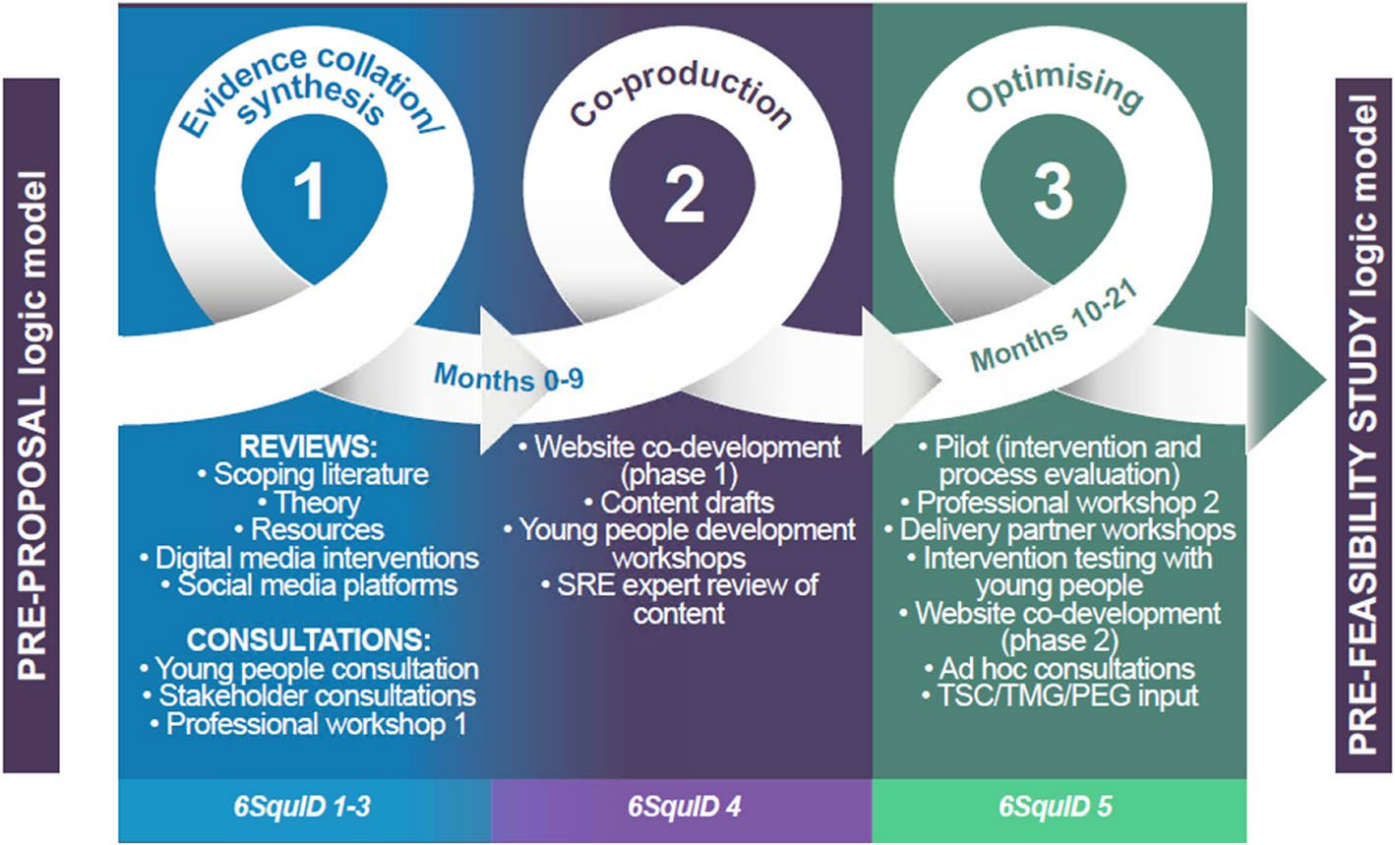
STASH Intervention Development Process (by 6SQuID component)

We began the intervention development process with a provisional programme theory, which visualised the problem, expected inputs, process of change, intermediate factors, and intended outcomes of the intervention (Fig. 1). STASH intervention development took 21 months, in three interconnected stages: 1) evidence collation and synthesis; 2) intervention co-production; and 3) adaptation (Fig. 2). The methods are described in detail elsewhere, [30, 32] and are thus only summarised here. Data production tools developed specifically for the study are included as Supplementary Files 1–3.

### Stage 1

#### Evidence collation and synthesis

Stage One comprised collation and synthesis of evidence intended to shape intervention content, delivery format, and the intervention’s overarching theoretical framework. It included an evidence review of relevant literature, theory, and resources, and consultations with stakeholders, including young people.

##### Evidence review

We conducted a comprehensive scoping review which, in brief, included: systematic reviews of related interventions; position papers; key literature on behaviour change theories (BCTs) with relevance to young people’s sexual wellbeing; and relevant theoretical literature on design and implementation of complex health interventions, including those drawing on Diffusion of Innovation Theory (which the basis of the ASSIST programme [21]).

Examples of high quality, youth-orientated SRE websites or resource packs, and policy and guidance on SRE delivery, were identified via the expertise of the TMG and targeted online searches. Data on source, type, quality, theoretical basis (if available) and key content were extracted. Resources were reviewed and rated for features, including relevance to the aims of STASH, quality, and perceived legitimacy. With respect to mode of delivery, candidate social media platforms were reviewed for user demographics and appeal, functionality, and regulatory information (primarily age restrictions)*.*

##### Consultations with stakeholders

We consulted with young people, teachers, and other stakeholders to explore proposed adaptations to the ASSIST delivery model and content, and potential local barriers to and facilitators of implementation. Consultation activities included qualitative interviews: group interviews with young people outside the subsequent feasibility study’s geographical area (two school-based and one community youth group, selected to represent a mixed range of socioeconomic backgrounds; 20 participants in total); and one-to-one interviews with four teachers involved in SRE. A semi-structured interview topic guide covered classroom SRE (including gaps), young people’s sexual health and behaviour, use of social media, and the proposed intervention format (see Supplementary File 1). Consultations also incorporated meetings with senior management teams from schools participating in the feasibility study, and Quality Improvement Officers/Child Protection Leads in participating Local Authorities. All activities were summarised and reviewed for recurrent themes, commonalities and differences, and key learning points.

We then held a workshop with 16 relevant peer education and youth work professionals, sexual health and health promotion specialists, academics, to develop intervention format and content. Small-group discussions reviewed key issues arising from the evidence review and the consultations noted above, and explored how to address these in STASH. The workshop also considered likely mechanisms of behaviour change and potential challenges for Peer Supporters (hereafter ‘PS’).

### Stage 2

#### Intervention co-production

Stage 2 comprised website co-development and design workshops with young people [31]. We used an iterative drafting process, building on the synthesis of evidence from Stage 1, to generate content for the STASH website and the PS training, organised by topic (e.g., relationships, consent, STIs). We worked with web development consultancy Antbits to produce a mobile-optimised interactive website for PS and trainers (including a content management system for use by the research team). Intervention training partners (specialist youth work organisations Fast Forward and West Lothian Drug and Alcohol Service (WLDAS) also collaborated closely on adaptions to the PS training manual and activities. An expert SRE educator reviewed and refined draft website content. Draft content was then presented to the two school-based groups who participated in Stage 1.

### Stage 3

#### Intervention adaptation

Stage 3 involved a pilot of the intervention in one school and refinement of the intervention for the feasibility study [19, 30, 32].

##### Intervention pilot

Over a nine-week period, we piloted the intervention in one school that was typical of schools in the feasibility study area in terms of size and socioeconomic factors. The accompanying process evaluation used a range of methods which we have detailed elsewhere, but which included: observation of PS training and student evaluation of training (at the start of the intervention, see Supplementary File 2); and an online questionnaire and semi-structured interviews with student participants, teachers, and trainers (and the end of the intervention – see Supplementary File 3 and [30, 32]). We also collated data from Facebook (group membership, number of messages sent) and project monitoring data (participation numbers, key contextual information, e.g., school interactions, contemporaneous events). Using an approach guided by the framework method [34], we reviewed findings in a matrix for commonalities, differences, and key learning to inform the feasibility study, regarding fidelity, acceptability, reach, recruitment/retention, and overall intervention sustainability.

##### Intervention refinement

A final series of workshops and meetings supported refinement of the STASH intervention for the feasibility study. The 16 professionals from the Stage 1 workshop attended a second workshop which reviewed findings from the pilot. We facilitated small-group discussions which focused on: the process of accessing and sharing from the STASH website; intervention reach, impact and sustainment; and PS training and follow-ups. Further refinements were identified through a series of ad hoc consultation meetings, including with: teachers and senior management teams at schools which would potentially participate in the feasibility study; and participants in the earlier professional panel; and intervention delivery partners [30, 32]. Website content was simplified and streamlined with the support of the [department] in-house graphic designer and Antbits.

Finally, we conducted semi-structured group interviews with two youth groups [30, 32]. Participants were aged 16–17 years and able to reflect on their preferences for information, mode of delivery at the intervention target age, as well as the relevance, relatability, and credibility of content created after the pilot.

## Results

The purpose of the STASH intervention development process was to assess and determine any necessary adaptations to the original ASSIST intervention model, including those that might support sustainability. The overall process took 21 months and included 20 activities across the three integrated stages. A full account of development work findings and intervention refinements derived from each stage are summarised detailed in the final report [30, Chapter 2 Appendix 1 – available here https://www.ncbi.nlm.nih.gov/books/NBK564691/]. Key findings are described by stage, below.

### Stage 1

#### Evidence collation and synthesis

Findings from our review suggested that, while young people might be well-informed about sexual risk, STIs and contraception, there were identifiable knowledge gaps in SRE provision, particularly around communication, consent, online (sexual health) literacy, and sexual pleasure [2, 30]. Our consultations with stakeholders and young people highlighted use of outdated SRE packages which did not cover topics such as consent and coercion. Young people also requested greater use of experiential information, humour, and interactive components.

We established that the support of schools would be indispensable to intervention implementation and sustainment. Consultations highlighted expectations on schools to meet national policy requirements (e.g., the Scottish Government’s Curriculum for Excellence), and thus foregrounded how STASH could support this and, in so doing, increase potential for intervention sustainability. Consultations also reaffirmed that the peer nomination component of ASSIST remained essential to ensuring PS credibility [20], and schools were requested not to influence this process. Professional stakeholders voiced concerns that both the older cohort and perceived sensitivity of the topic would make intervention delivery difficult and queried whether PS would be mature enough to manage their role. It was also suggested that it could be challenging to recruit the 15% required for effective intervention diffusion throughout the year group [20], given the topic and age group.

**Fig. 3:**
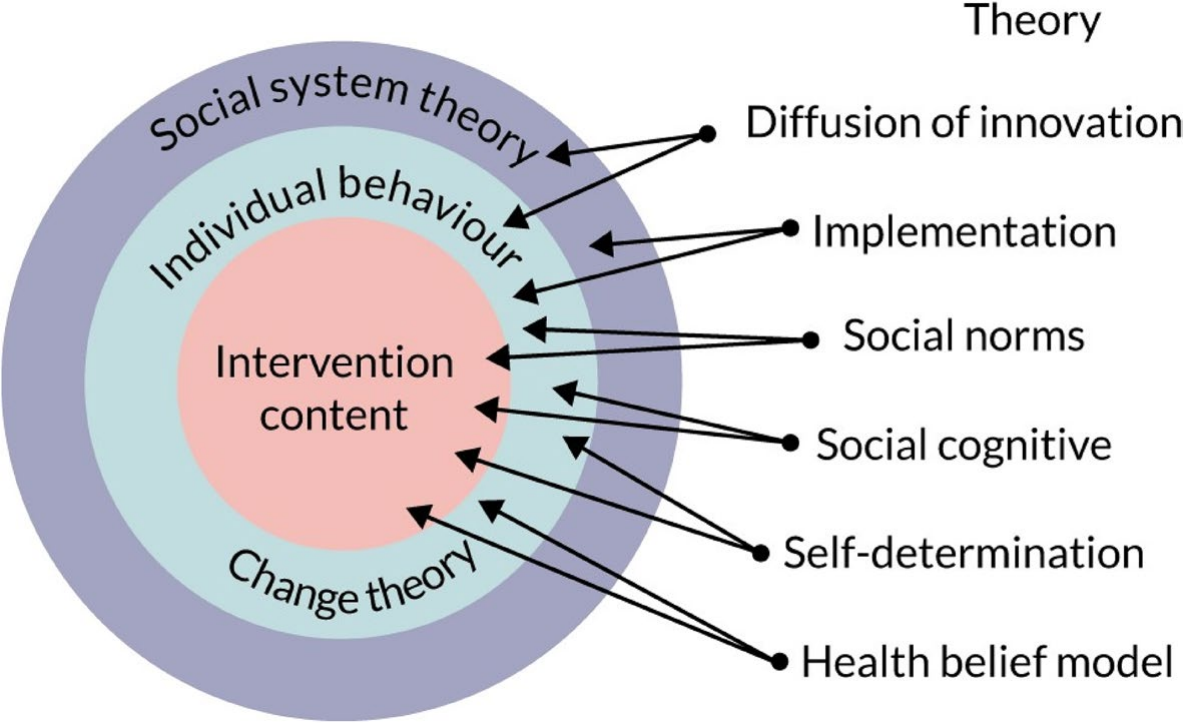
Theory used in STASH intervention development

Through review of the theoretical literature, we identified the most relevant theories to inform the mechanisms of change at the levels of the social system of the intervention, individual behaviour change, and the specifics of intervention content. Diffusion of innovation theory [21], which was the basis for ASSIST, provided the overall theoretical grounding for STASH, while implementation theory [35] identified the particular elements on which the intervention should build to optimise sustainability. These were further supported by specific behaviour change techniques derived from social norms theory, social cognitive theory, self-determination theory, and the information, motivation and behaviour model [36–39] (see Fig. 3). Drawing on these, we established a need to prioritise skills development—particularly around communication – and to support both active participation and responsibility-taking, in the intervention itself, and in the context of sexual behaviour. Our findings also suggested a need to enhance self-efficacy and intrinsic motivation; and to tailor all intervention content to the needs, life stage, and key concerns of the target group.

Our review of online resources foregrounded the existence of a number of training activities and websites for young people. We shortlisted those resources that best reflected both the content requested by young people during the consultation; and effective utilisation of the intervention theories described above. Current relevance of resources varied. Some were designed for national use in formal education, some driven by individual practitioners, and others were topic specific. Most included communication, relationships, bodies, STIs, but not all included online issues, porn, sexting, and consent. Some topics – such as sexual function, pleasure, accurate anatomy of female genitalia – were almost entirely absent. Each included some materials which could be adapted for inclusion in PS training and follow-up sessions. The BISH website (www.BISHuk.com) appeared to have the most comprehensive content and engaging (mixed media) format, though it was recognised as potentially more frank in tone than might be acceptable to schools. A vast array of highly-viewed, often user-generated digital media sources – such as YouTuber content – were also identified as suitable for inclusion.

The most popular and commonly used social media platforms for the target group at that time (2016–17) were identified as Instagram, Snapchat, WhatsApp, Facebook, Twitter, Pinterest, Tumblr, and Vine. All but Facebook were ruled out due to restrictions in functionality. Facebook was the only platform meeting intervention requirements identified in earlier stages, namely: to enable an interface with an external website and the posting of web links, images, and text; create private, invite-only groups, which could be monitored (for research and safeguarding purposes); and provide ease of viewing/message stability over time.

### Stage 2

#### Intervention co-production

##### Intervention content

Taking ASSIST training activities as a basis, new intervention content was tailored to the new topic and relatively older age group (14–16, rather than 12–13-year-olds). Topic-specific content for the STASH website, PS messages, and intervention training materials were also generated. These drew on the key themes from the Stage 1 evidence review and retained much of the generic communications skills training from ASSIST. Testing with young people suggested that memes and images or videos, straightforward language, and the experiences of young people were preferred components. Co-development workshops and SRE expert review of the website and training content also highlighted a preference for minimal text, and for inclusive content, to reflect and respect the spectrum of gender and sexual identities.

##### Adaptations from ASSIST

STASH retained the key mechanisms of change from ASSIST, with Diffusion of Innovation Theory remaining the theoretical core, along with recruitment of ‘early adopters’, and the use of professional trainers to support PS. (See supplementary file 4 for the recruitment/peer nomination questions used.) In ASSIST, PS completed pro forma diaries to track activity, but entries were not always accurate, and some missing altogether [40]. The use of Facebook for message sharing in STASH groups (to which the research team were granted access) allowed for some PS activity to be monitored directly. Numbers of participant-reported face-to-face PS conversations were recorded by trainers at weekly follow-ups. To address schools’ early concerns about potential online bullying, and how PS would cope with sensitive disclosures, a trainer was included as a member of all PS Facebook groups. We also added a ‘STASH charter’, which reiterated the values and expectations of the role, and which PS signed up to on completion of training. We included specific training on sensitive disclosures, noting the need for referral to a relevant adult, as appropriate. We emphasised to PS that it was not their role to provide counselling, beyond what they would typically discuss with friends.

### Stage 3

#### Intervention adaptation

##### Pilot study

We present key findings from the pilot stage in relation to the primary issues identified and subsequent refinements made.

31 of 163 students in the year group were nominated and invited to the PS recruitment meeting. Of these, 19 were trained, and 14–17 attended each follow-up session (attendance varying weekly). None explicitly withdrew.

PS were engaged in the training, though there was some embarrassment, and observations highlighted visible discomfort with roleplays activities. Trainers generally perceived the group to have coped well with the training.

I just think their nature, they're outgoing. They maybe were a wee bit shy to start wi' but they've settled into a good group. The group dynamics are just right. They're very mature. Aye, alright, you get the odd bit o' immature [behaviour], but I thought there would've been a lot more, credit to them. (Trainer interview)

Friends of PS who were interviewed did not think all PS were necessarily right for the role and suggested they would have nominated others if they had known the subject matter. They suggested some PS had not taken the intervention seriously, perhaps lacked confidence, or just did not share details of the project beyond the PS group.

Int: Do you think that the people that were asked to be peer supporters then were kind of the right people to pick?

Friend1: Yes. I only know…

Friend2: Aye, like some o' them, yes. Some o' them, no. Bit o’ a mixture.

Friend3: Like, there is some people in the year that I think would get right into it.

Friend1: Benefit fae [from] it.

Friend2: Uh huh, and would come and speak to you about it. But, like, the people that I think are doing it, 'cause they’re like your friends would come up to you and just be like “Oh, this is what we done today” and that's it. They wouldn’t really go into detail about it. (Friends interview)

That all PS in the pilot came from two large, overlapping friendship groups may also have been a limitation to intervention reach (and an idiosyncrasy of the pilot year group). This highlighted that some characteristics of existing social networks in the target group could potentially hinder, as well as support, diffusion.

The pilot also highlighted practical issues which impacted recruitment, including the need for further time to distribute and return parent/carer consent forms. Training evaluation data and PS questionnaire findings suggested that potential CV benefits, getting to spend time with friends, and a sense of responsibility/satisfaction in nomination made the role attractive, meaning promoting these could increase uptake. Overall awareness of STASH was low among non-PS S4 students and teachers, suggesting profile-raising activities should be considered.

Most PS (74%) shared three or more messages but few reported face-to-face conversations. PS often posted after prompting via trainer posts. Incidental data captured in the project log suggested that the required login and limiting use to PS rather than the wider student group, created an unnecessary barrier to using the STASH website. PS reported finding the trainer’s presence in the Facebook groups reassuring, but that they did have concerns about sharing messages with students outside their immediate friendship group. This highlighted that more emphasis may have been needed at the training stage on the expectation that PS need only interact as far as they were comfortable within their existing friendship groups.

##### Subsequent refinement

In a final series of development activities following the pilot, implications of the findings and possible intervention refinements were discussed and agreed with the professional stakeholders and young people. Discussions focused on how to broaden recruitment and reach, and increase engagement with the intervention for PS, other students, and teachers. Consideration of the limitations of the piloted website design informed the decision to revise the content and to make it available to all students, not just PS. We explored gamification of online elements of STASH to enhance interactivity and engagement, but ultimately dropped this to prioritise simplicity in the resources. Some suggested additions (e.g., a search function and ‘escape’ button, to allow the site to be quickly closed) were beyond our resources. We streamlined sub-sections, increased the number of bespoke infographics and memes, and removed most external links and all conversation prompts (the latter having been largely unused). The revised website tested well with young people.

### Overall intervention refinements and the revised STASH programme theory

**Fig. 4:**
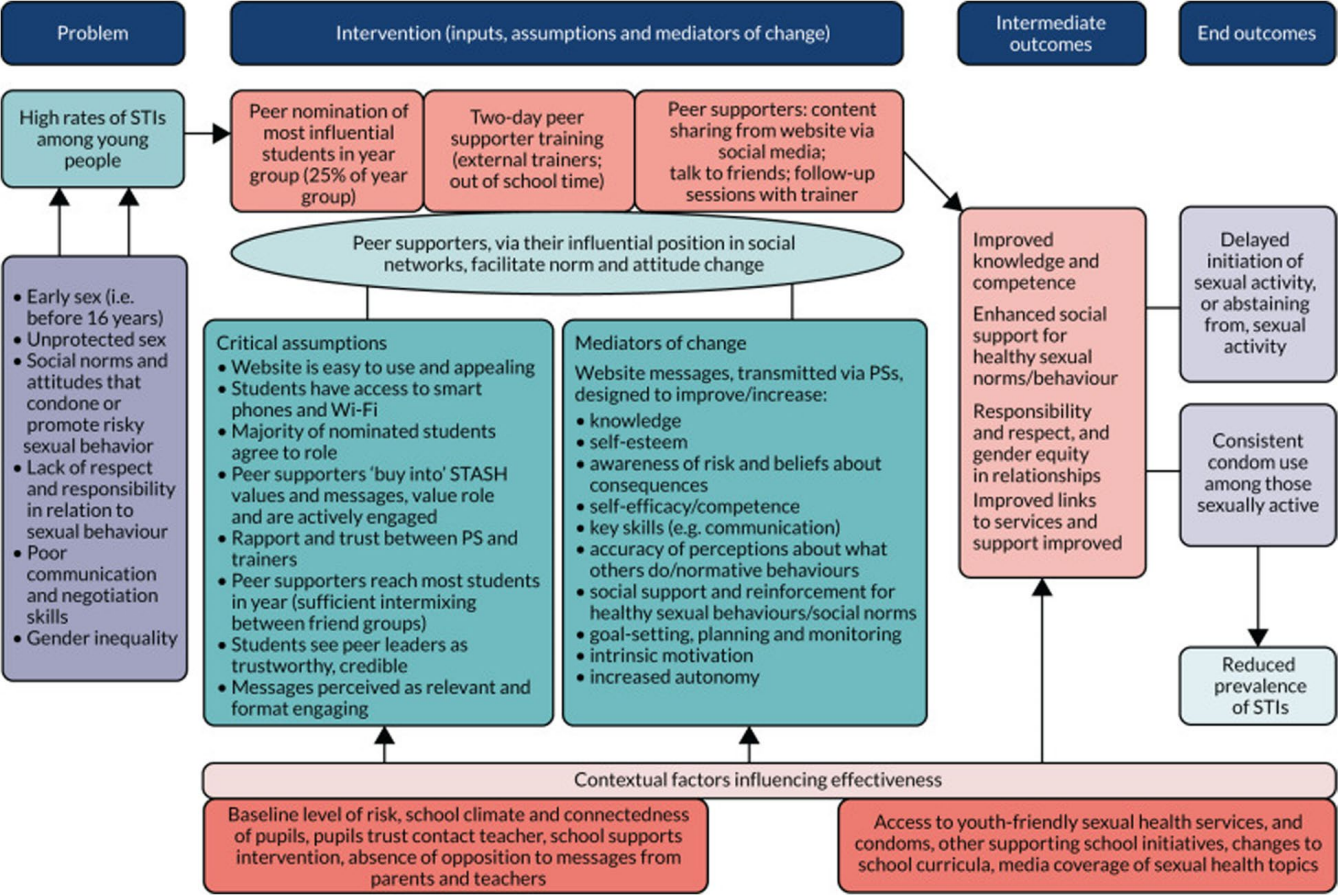
STASH Programme Theory v2.0 (End of development process – post-pilot, pre-six-school feasibility study)

The above process produced a revised STASH intervention and accompanying programme theory. The post-pilot intervention design is detailed elsewhere [32]. Figure 4 shows the revised programme theory. Key intervention adaptations and overall refinements are summarised below.

#### Overall intervention design

The development process suggested we should maintain the six overarching mechanisms from the ASSIST model: nomination, recruitment, training, peer supporter-delivered activities, trainer-led follow-ups, and participant acknowledgement. We also retained three of the six original ASSIST nomination questions (pertaining to ‘respect’ and ‘leadership’). To tailor and enhance nomination of the most appropriate and effective PS, we added four new questions for STASH, devised to address to whom students would feel comfortable talking about sensitive issues, and who was perceived as encouraging, trusted, and confident. Since the 12% recruitment rate in the pilot fell short of the 15% ‘critical mass’ identified in the ASSIST trial as necessary for diffusion of innovation [20], we increased the percentage of student nominees that would be invited to the recruitment meeting in the feasibility study to 25% of the year group (rather than 17.5% recommended in ASSIST). We asked the contact teacher and PS to agree how to let the wider year group know about STASH to increase awareness. In the main feasibility stage, and following the suggestion of one PS, we distributed STASH PS badges to PS to support other students in identifying them.

##### Website and online activities

Development work findings resulted in a website significantly revised from the initial iteration. To maximise reach, particularly with Facebook non-users, PS were given STASH cards (‘business cards’ with the STASH logo and web address) with which to promote the project. It was agreed that peer supporters should be asked to establish ‘secret’ (invite-only groups; highest privacy setting) Facebook groups – comprising friends and the STASH trainer account – in which the intention would be that they share messages from the STASH website. A significant amount of work went into producing visually attractive, concise content, which could easily be shared from the STASH website to the Facebook groups.

##### Peer supporter training and role adaptations

Adaptations here included prioritisation of smaller group work and streamlining of topics and activities. Activity types with which PS were less comfortable were removed or revised. To increase confidence, and support PS in establishing and posting in a ‘secret’ Facebook group, we integrated the website and use of Facebook into the training to a much greater degree. PS-delivered activities were explicitly clarified to focus on: giving information; influencing attitudes; promoting the STASH website; and signposting friends to trusted adults/sexual health services. Training and follow-up sessions revisited these components a number of times. As STASH sought to address a complex set of issues and behaviours, we decided to include one additional follow-up session at the feasibility study stage, resulting in a total of five sessions. Finally, multiple methods of PS acknowledgement were devised, including a ‘Peer Supporter of the week’ prize (a STASH-branded highlighter pen), and a University of Glasgow certificate. Schools were also encouraged to support PS who wished to use their involvement in STASH to work toward a Saltire Award (https://saltireawards.org.uk/).

## Discussion

We have detailed the comprehensive and rigorous process taken to intervention development in STASH, which incorporated three iterative stages of evidence collation and synthesis, co-production, and adaptation. Findings from each stage were used to optimise the intervention design for the subsequent six-school feasibility study and revise the programme theory [30, 32]. This paper offers insight into the depth and scale of co-development work required to adapt and refine the intervention from the original ASSIST model, and to ensure it was appropriate for a different topic and behaviours (sexual health instead of smoking) and age group (14–16 instead of 12–13 years). In the remainder of this paper, we reflect on the utility of the intervention development frameworks used, key strengths and limitations to our approach, and the challenges of co-production in the context of a school-based young people’s sexual health intervention.

Combined use of the MRC guidance on intervention development, the 6SQuID framework, and Hawkins’ et al.’s framework (co-production, prototyping) supported us in addressing key considerations in developing a young people’s sexual health intervention. These considerations included integration of theory; consultation and co-production; and an adaptation and refinement phase. Addressing these helped to ensure that our adaptations to the ASSIST model were theoretically grounded, practically applicable, and implementable. Crucially, the intervention tested subsequently has since been evidenced as feasible [30, 32] in a way that others have not, an outcome arguably supported by our rigorous approach to adapting and optimising a pre-tested model. Our approach also echoes the INDEX best practice guidance [41]. The adaptation phase was also beneficial in highlighting potential issues around acceptability and overall feasibility of the intervention to be explored further in the six-school feasibility study [30, 32]. Side-stepping this extensive, iterative process risks development of a sub-optimal intervention, failure at the implementation stage or, ultimately, failure of wider adoption and embedding [25]. While sustainability of implementation is notoriously difficult to assess [42], our approach goes some way to supporting sustainment from the outset. To complement theoretically sustainable interventions, however, the sexual health and wellbeing needs of young people need to be prioritised at a policy and resource level.

The STASH intervention development process was complex, comprehensive, rigorous, and grounded in co-development and participatory mixed methods. It benefited from broad engagement with experts in the topic area and implementation context and, most importantly, with the target population of young people. Close collaboration with an appropriate web developer, and with our training providers (Fast Forward and WLDAS) provided specialist expertise, and was thus a particular strength of this study. We also found the iterative process of developing and visualising an overall programme theory to be beneficial not only in understanding how the theories drawn upon were applied in practice, but also in communicating the intervention’s purpose and key elements to stakeholders and implementers.

Our approach was time- and resource-intensive, with multiple activities conducted over almost two years. Rather than being a limitation, however, this was the breadth, depth and scale of activities required for a comprehensive approach, and is hence another key strength of our intervention design. We relied on rapid review methods in our evidence synthesis and some sources could have been excluded or missed. We would suggest that the balance of evidence review and stakeholder consultation makes this unlikely, as review findings were presented to and built on by our stakeholders in discussion and development sessions. The STASH intervention has been developed in the Scottish context, but we see no reason for it not to be applicable further afield, with appropriate attention to transferability across contexts [43].

One key limitation lay within the approach taken to social media use, and the use of Facebook in particular. We were limited both by the resources available and the technical specifications of social media platforms. (Facebook was the only social media platform that met the intervention requirements, while more popular platforms such as Snapchat, WhatsApp, and Instagram did not.) While Facebook use among younger people may broadly be declining [31], consultations at the time suggested that at the time Facebook was still widely used and acceptable to young people for the purposes of a project like STASH. Full embedding of interventions like STASH in the digital lives of young people requires ongoing attention, however, and resources should be factored in to enable platform flexibility in particular. The mismatch between the rate at which social media evolves versus that at which it is feasible to conduct rigorous social scientific intervention development makes it extremely challenging for publicly funded research to keep pace and maintain currency. We explore these and other issues – including the question of the ‘critical mass’ of peer supporters required in this context—in relation to the main feasibility trial elsewhere [30, 32].

One further challenge which emerged in this development work lay in reconciling theoretical elements and intervention design features with the needs and concerns of the target group (young people) and key stakeholders (particularly schools and those in the relevant local authorities with responsibility of child protection), in a way which was practically workable. This was particularly pronounced in the process of co-production, where tensions among the priorities of each group and with maintaining a workable theoretical grounding, were most evident. For example, a key learning point for us lay around relaxing ‘control’ over implementation (e.g. removing the need for website login credentials in order to broaden access) in way which allowed more flexible engagement at the feasibility study stage. At the pilot stage, we arguably constrained the potential mechanisms of change in our efforts to allay stakeholder concerns (concerns which were ultimately not evidenced in the pilot).

Co-production can maximise acceptability, feasibility, and sustainability, and generate ownership and buy-in [26]. These are particularly acute concerns in the resource-tight context of schools, and for a target group of young people who might quickly deem the tone of an intervention to be patronising or unappealing. Early and meaningful involvement of young people in developing sexual health research is crucial [44]. Incorporating the sexual health information young people really want in a school-based social media-related setting is extremely challenging: significant considerations have to be navigated around safeguarding concerns, the ‘sensitivity’ of the subject matter, and questions around what interactions an intervention might encourage young people to have, and with whom. This also has to be balanced with the resources available to those developing the intervention. However, these complexities and challenges highlight why it is all the more essential to draw on what is known to work, rather than perpetually reinventing the intervention wheel.

## Conclusions

The comprehensive process of STASH intervention development—which incorporated three iterative stages of evidence collation and synthesis, intervention co-production, and adaptation—enabled us to tailor an effective existing intervention to a new topic and context. This process maximised the likelihood that the version of the intervention subsequently tested would be acceptable, feasible and effective in the round. This is crucial to demonstrate before significant resource is invested in further testing. Our process provides a rigorous model for future intervention development, which can serve as an example for developers, researchers, policy makers and practitioners not only in sexual wellbeing but other areas of health more broadly.

## Supplementary Information

**Additional file 1: Supplementary file 1.** Development Interview Topic Guides, Topic guides for development interviews with teachers and students.

**Additional file 2: Supplementary file 2.** Pilot Training Observation and Evaluation, Semi-structured observation guide (pilot version used in development work) peer supporter training and evaluation forms for students and accompanying teachers.

**Additional file 3: Supplementary file 3.** Pilot Peer Supporter Questionnaire, Online questionnaire (pilot version used in development work) for peer supporters following the pilot intervention.

**Additional file 4: Supplementary file 4.** Peer nomination questions.

**Additional file 5.** GUIDED checklist, reporting checklist for STASH intervention development work.

## Data Availability

The relating to this paper and the subsequent feasibility study may be requested via DOI:10.5525/gla.researchdata.1110.
