# Supplementary material for: A peer-led, school-based social network intervention for young people in the UK, promoting sexual health via social media and conversations with friends: intervention development and optimisation of STASH
Source: BMC Public Health. 2023 Apr 11;23:675. doi: 10.1186/s12889-023-15541-x (PMC10088210; doi:10.1186/s12889-023-15541-x)
Supplement: Supplementary file 2 — Additional file 2: Supplementary file 2. Pilot Training Observation and Evaluation, Semi-structured observation guide (pilot version used in development work) peer supporter training and evaluation forms for students and accompanying teachers. [file 12889_2023_15541_MOESM2_ESM.docx]

Supplementary file 2 - Pilot Training Observation and Evaluation, Semi-structured observation guide (pilot version used in development work) peer supporter training and evaluation forms for students and accompanying teachers.
