# Supplementary material for: A peer-led, school-based social network intervention for young people in the UK, promoting sexual health via social media and conversations with friends: intervention development and optimisation of STASH
Source: BMC Public Health. 2023 Apr 11;23:675. doi: 10.1186/s12889-023-15541-x (PMC10088210; doi:10.1186/s12889-023-15541-x)
Supplement: Supplementary file 3 — Additional file 3: Supplementary file 3. Pilot Peer Supporter Questionnaire, Online questionnaire (pilot version used in development work) for peer supporters following the pilot intervention. [file 12889_2023_15541_MOESM3_ESM.docx]

Supplementary file 3 - Pilot Peer Supporter Questionnaire, Online questionnaire (pilot version used in development work) for peer supporters following the pilot intervention.
