# Supplementary material for: A peer-led, school-based social network intervention for young people in the UK, promoting sexual health via social media and conversations with friends: intervention development and optimisation of STASH
Source: BMC Public Health. 2023 Apr 11;23:675. doi: 10.1186/s12889-023-15541-x (PMC10088210; doi:10.1186/s12889-023-15541-x)
Supplement: Supplementary file 4 — Additional file 4: Supplementary file 4. Peer nomination questions. [file 12889_2023_15541_MOESM4_ESM.docx]

Supplementary file 4 - Peer nomination questions.
